# Genetic Programming Based Ensemble System for Microarray Data Classification

**DOI:** 10.1155/2015/193406

**Published:** 2015-02-25

**Authors:** Kun-Hong Liu, Muchenxuan Tong, Shu-Tong Xie, Vincent To Yee Ng

**Affiliations:** ^1^Software School of Xiamen University, Xiamen, Fujian 361005, China; ^2^Department of Computing, The Hong Kong Polytechnic University, Hung Hom, Kowloon 999077, Hong Kong; ^3^Baidu Inc., Beijing 100000, China; ^4^School of Computer Engineering, Jimei University, Xiamen, Fujian 361021, China

## Abstract

Recently, more and more machine learning techniques have been applied to microarray data analysis. The aim of this study is to propose a genetic programming (GP) based new ensemble system (named GPES), which can be used to effectively classify different types of cancers. Decision trees are deployed as base classifiers in this ensemble framework with three operators: Min, Max, and Average. Each individual of the GP is an ensemble system, and they become more and more accurate in the evolutionary process. The feature selection technique and balanced subsampling technique are applied to increase the diversity in each ensemble system. The final ensemble committee is selected by a forward search algorithm, which is shown to be capable of fitting data automatically. The performance of GPES is evaluated using five binary class and six multiclass microarray datasets, and results show that the algorithm can achieve better results in most cases compared with some other ensemble systems. By using elaborate base classifiers or applying other sampling techniques, the performance of GPES may be further improved.

## 1. Introduction

With the development of microarray technology, it is possible for one to measure the expression levels of thousands of genes simultaneously. Although it provides a gold mine of biological information and knowledge, it brings new challenges for biologists, statisticians, and machine learning researchers because of the high dimension and small sample size problem. In general, classification models can deal with more features only when enough samples are provided; otherwise the model is very likely to overfit the training data. In other words, for microarray classification, the difficulty of bias-variance dilemma [1] is mostly on the variance side.

Many researchers are devoted to designing new learning models for classifying different diseases based on microarray data, and there are already many successful applications. In such cases, some techniques are widely used in models, such as feature selection and regularization techniques. Among them, an effective scheme to reduce overfitting is ensemble learning. In general, ensemble learning requires a set of base classifiers, which are then combined to form final decisions instead of trusting only the best one. This technique has been proved to be able to boost accuracy significantly in many fields. Clearly, ensemble learning cannot improve the results of single predictors if all the base classifiers were the same. On the other extreme, generating totally random base classifiers (without training) would totally fail anyway because their performances would be close to that of a random guess. As pointed out in [2, 3], the improvement of ensemble learning is determined by a balance between accuracy and diversity of base classifiers.

For classification problems, we can denote the *N* training samples by (*x* ^1^, *y* ^1^), (*x* ^2^, *y* ^2^),…, (*x* ^*N*^, *y* ^*N*^), where each *x* is a *M*-dimensional vector and each *y* is a scalar in {1,…, *c*} for a *c*-class problem. Diversity can be injected at different levels: sample, feature, and base classifier: (1) for injecting diversity into samples, one does not need to alter the feature space of *x*, but the distribution of samples. Typical methods include Bagging [4] and Boosting [5]. In Bagging, base classifiers are trained with bootstrap training samples; thus, each base classifier is expected to deal with different training sets. In Boosting, the base classifiers are generated by iterations. And in each iteration, weights of samples are adjusted, so that samples misclassified by current trained classifiers are emphasized, and the following classifiers can concentrate on them. (2) For injecting diversity into features, one maps the original *M* dimensional feature space into a *M*′ dimensional space. When the size of original feature set is relatively small, some researchers may try to add some combined features to make data more informative, resulting in *M* < *M*′. On the contrary, when *M* is large, such as in the case of microarray data analysis, researchers may tend to apply feature selection techniques to ensure *M* > *M*′, so as to simplify the problem. Ho [6] and Bay [7] trained multiple base classifiers with random subset of features, and this technique was named as “random subspace” [8]. Bryll et al. [9] came up with the attribute Bagging method, which was proposed based on the combination of Bagging and wrapper feature selection. Random Forest was introduced by fusing the ideas of Bagging and random subset of features [10]. And Rotation Forest model was proposed by using feature extraction methods on different sample and feature subsets, so as to generate diverse rotation matrices for base classifiers [11]. In this way, each classifier rotates the original feature space in different directions. (3) For injecting diversity at the level of base classifiers, an intuitive scheme is to train base classifiers with different parameters. A typical application is that Maclin and Shavlik used a competitive learning scheme for training neural networks with diverse initial weights [12]. The mixture of different types of base classifiers can also be an effective method to produce diverse ensemble systems. For example, one can use SVM, neural network, and decision tree at the same time in an ensemble system. In addition, it is also applicable to inject diversity at different levels in the process of building ensemble systems. For example, Kim and Cho [13] used different feature selection methods (at feature level) and classifiers (at classifier level) and applied an evolutionary algorithm to find a group of optimal combinations of feature-classifiers pairs. The combination of different levels can further improve the diversity in general.

After generating *L* base classifiers, the next key to producing a powerful ensemble system is to select a subset from them as the final committee in a static or dynamic way, which is referred to as the ensemble selection problem. When *L* is small, we usually just keep them all. However, when *L* is set to a large value, it may be redundant and even ineffective to keep all of available base classifiers. For example, if a large proportion of these base classifiers are similar or correlated, the opinions of the minority would be ignored, and the diversity of the ensemble committee cannot be guaranteed. The simple strategy of selecting the top *K*  (*K* < *L*) base classifiers also faces this problem, because the top base classifiers may turn out to perform similarly. Another feasible strategy is to use some diversity measures to guide the search. However, the usefulness of diversity measures is doubtful [3]. Usually, when there are many samples in each class in a dataset, researchers can divide it into three independent sets: training, validation, and test sets. The work of Ruta and Gabrys [14] indicates that the best criterion is the combined accuracy on a validation set, at least when majority voting is selected as the fusion strategy. However, for the microarray data analysis problem, the whole sample size is so small that there are less than 100 samples in each class in most cases. As a result, almost all microarray datasets contain only training and test sets. An independent validation set is completely unaffordable, which makes the analysis of microarray data a tough task.

In this paper, we propose a genetic programming (GP) based ensemble system (GPES for short) to tackle this problem. GP is a widely deployed evolutionary algorithm and has been successfully applied in many research fields. It can be used to generate decision rules for binary class classification problems directly, because each individual in GP is a syntax tree, which can produce a “yes/no” answer. So in the field of microarray analysis, Langdon and Buxton [15] and Yu et al. [16] used GP to fulfill the tasks of cancerous gene selection and classification model generation simultaneously. Besides, Hong and Cho [17] proposed a diverse ensemble of classifiers with individual rules generated by GP. And we extended the original GP framework by designing a new individual structure (named as subensemble system) to deal with multiclass problems directly [18]. However, in all these cases, it is a quite time-consuming task to use GP to generate classification rules due to the computational complexity. As a result, these GP based algorithms are generally slower than elaborate classifiers, such as kernel based SVM and neural networks.

On the contrary, the usage of GP in this work is different from those introduced above. It is used to fuse base classifiers and produce robust ensemble systems. In the framework of GP, each individual is a single tree, representing a set of possible compositions of functions and terminals selected from the nonterminal and terminal sets. In our algorithm, the terminals are decision trees, and nonterminals are fusion operators (Average, Min, and Max in our algorithm). GP begins with random initialization, and it evolves towards the goal of low classification error rate. And as pointed out in [19], different fusion strategies can guarantee diversity, so the individuals generated by GP could be of high diversity and low error rate in the evolution process. A number of individuals in the last generation of GP are selected with a forward search technique to form the final ensemble committee. In this algorithm, diversity is introduced at all of three levels. So the final ensemble consists of diverse and accurate members. And the experiments in different microarray datasets verify the high generalization ability of our method. Decision tree, as a kind of preferred model for many ensemble frameworks (Bagging, Random Forest, Rotation Forest, etc.), is deployed as the base classifier in our algorithm because it is an unstable model [20]. It should be noted that although our ensemble system only employs decision trees, other classifiers, such as SVM and neural network, can also be used in this framework. Some earlier works in this paper have been presented at ICIC 2014 conference [21].

## 2. Methods

### 2.1. The Principle of Genetic Programming

**Figure 1:**
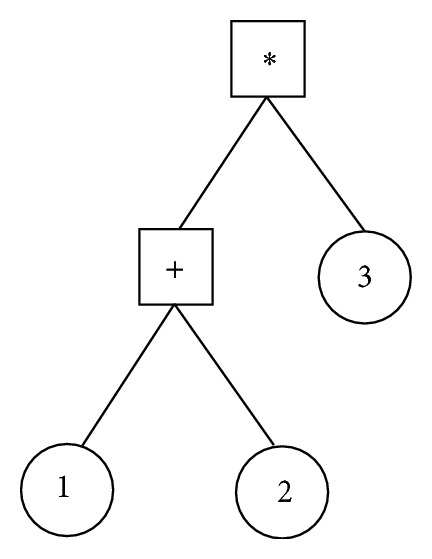
A simple syntax tree for arithmetic.

GP is a widely used evolutionary algorithm, and it has been proved to be an effective solution for many optimization problems. Essentially, GP is a branch of genetic algorithm (GA), and the main difference between GP and GA is the structure of individuals: GA has string-structured individuals, while GP's individuals are trees, as shown in Figure 1. There are two kinds of nodes: terminal and nonterminal nodes. A terminal node is a leaf node without child nodes. And a nonterminal node is an inner node with child nodes, which can be terminals or nonterminals. Usually, terminals are primitive elements, and nonterminals are operators for combining these primitive elements. Figure 1 illustrates a simple syntax tree for arithmetic, where nonterminals (×, +) are surrounded by rectangles, and terminals (numbers) are surrounded by circles.

Just like GA, there is a pool of individuals that compete with each other in GP. The initial population is usually generated randomly. A fitness function is designed to evaluate the performance of each individual. And only the individuals with higher fitness values are selected to survive and produce offspring. After that, crossover and mutation operators are applied to the surviving individuals. The purpose of the crossover operator is to create a chance of integrating strengths of different individuals, which is usually done by swapping some branches of two individuals (trees). The purpose of the mutation operator is to inject randomness to avoid falling into local minima in the evolutionary process. And it can be achieved by changing some terminals or nonterminals of individuals randomly. After finishing the process of crossover and mutation operations, some new individuals are produced to join the surviving individuals, so as to form a new generation. And this process keeps on iterating until a criterion is met. As a result, the population moves towards the global optimum rapidly.

### 2.2. Growth of Decision Trees

Decision tree is widely used in different ensemble systems, such as Bagging, Random Forest, and Rotation Forest. Its advantage lies in that it is an unstable model, and even small perturbations in inputs can cause great difference among the trained trees. This can reinforce the diversity among the base classifiers, which is important for an ensemble system. It is deployed as base classifiers in our algorithm either, making each individual of GP be an ensemble of decision trees. Each terminal is a single decision tree, and each nonterminal is one of the fusion operators: Average, Max, and Min. In the binary class problem, let the label be −1 for the negative class and +1 for the positive class. Then the Min operator prefers the negative class. That is, if one of its child nodes outputs a negative vote, the final decision of the Min operator is a negative label (−1) even when more children produce positive votes. The Max operator works in a diametrically opposite way, and the output is positive class label if one of its children outputs positive label (+1). Unlike the regular definition of “average” in mathematics, the output of Average operator is a hard label. That is, when the mean value of inputs is smaller than 0, then the Average operator regards the final vote as a negative label (−1); otherwise, it outputs a positive label. So the Average operator works in the same manner as the majority voting, and the final decision is the label obtaining the most votes. Because of the small sample size problem, if the accuracies on training sets are used as weights for base classifiers, the algorithm may be confronted with the overfitting problem. So for the Average operator, all votes are treated as equal.

**Figure 2:**
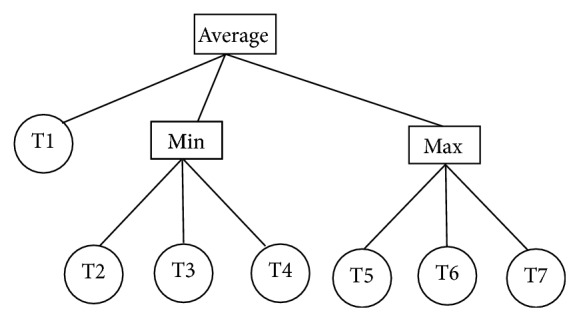
An example of the individual of GP in the proposed algorithm.

In order to make these operators work effectively, each nonterminal is set to contain three children, which can be either terminals or nonterminals. An example of an individual is illustrated in Figure 2. Here, T1–T7 are decision trees, and Average, Min, and Max are fusion operators. In this individual, if T1, T2, and T5 produce negative votes for a sample and others produce positive votes, the final output of the ensemble system is a negative label.

The process of building decision trees is described as below.

#### 2.2.1. Feature Standardization

Before training, each feature is standardized to zero mean and unit variance across training samples, and the same standardization step (fitted from the training set) is also applied to test datasets.

#### 2.2.2. Feature Subset Selection

Microarray datasets consist of large amounts of features, most of which contain little information. As pointed out in [22], usually a small number of genes are enough for the purpose of classification. Thus, to speed up the training process and boost the accuracy, feature selection is often applied before the training phase.

It was found that the use of various feature selection methods benefits experimental results greatly [23]. It is because different feature selection methods are based on different assumptions, and the biologically significant feature subsets would be picked with higher probability by combining the results of different feature selection methods. So we apply the following four popular feature selection methods:

- *F*-Test: it is a classical technique used in one-way analysis of variance, and it tests whether the population means of different groups are equal. We use the implementation in scikit-learn library (0.12) [24] with the default setting;
- RELIEF: it utilizes the nearest neighbor classifier to evaluate the significance of features [25] and has been successfully applied in microarray data classification [26]. We use the implementation in mlpy library (3.5.0) [27], setting the parameter “*sigma*” to 2, as suggested in [26];
- Random Forest: the quality of splitting classes in decision trees can be used for assessing the importance of features. So Random Forest is used for feature selection by summing up the scores assigned by all decision trees. We use the implementation in scikit-learn library (0.12) with the default setting;
- SVM-RFE: this is an embedded feature selection method. It iteratively trains SVM and removes a number of the least important features [22]. We use the implementation in scikit-learn library (0.12) using linear kernel. The parameter “*step*” is set to 2 for recursive feature elimination (RFE). In this way, there are two features to be removed at each iteration. Other parameters are set by default.

Each of the feature selection methods selects 50 features, and the combination of all features is kept to form a pool of candidates without duplicates. In this way, the dimensionality of datasets is reduced to a number within the range [50, 200]. After that, we adapt the idea of random feature subset selection, so that each decision tree only sees a random projection of the candidate pool. For each tree, *N*_*f*_ features are randomly selected obeying a Gaussian distribution, setting the mean to 5 and the standard deviation to 3. Because each tree grows with only a small proportion of the features, base classifiers can be quite different. As a result, the diversity of the ensemble system is maintained at the feature and base classifier level. In addition, there are only five features used to train decision trees on average, so the scale of each tree is limited. Since each individual contains a group of decision trees, the small scale of each tree can guarantee the efficiency of our ensemble system. The base classifiers with poor performances would be filtered out in the following process.

### 2.3. Balanced Subsampling

In the process of growing decision trees, we adapt the idea of Bagging to encourage diversity at the level of samples. As originally described, Bagging randomly selects sample with replacement to introduce diversity. However, for microarray datasets, we usually do not have many samples; thus, sampling with replacement cannot ensure diversity in training datasets. Another concern is that in most cases microarray data are unbalanced, which usually reduces the generalization ability of a classifier.

Taking these two factors into consideration, we used a balanced subsampling technique. For two-class classification problems, the numbers of samples in the two classes are denoted by *N*_1_ and *N*_2_, respectively. The number of samples in the smaller class is *Ns* = *Min*⁡(*N*_1_, *N*_2_). When building a classifier, *Ns* samples are sampled without replacement in each class. Thus, all the samples in the smaller class are kept, while the samples in the larger class will be subsampled without replacement. In the GPES framework, multiclass problems are divided into a set of binary class problems, and this subsampling technique is also applied to these binary class problems.

### 2.4. Evolutionary Process and Ensemble Selection

Based on the tree building process described above, 300 decision trees are firstly generated as candidates. Due to the random feature subset technique, there inevitably exist decision trees with poor performance. To solve this problem, only the trees with above average accuracy among the candidates are selected to construct a pool of base classifiers. In this way, only accurate classifiers are kept in the pool, which is used as the set of terminals in GP.

After that, a typical GP evolution schema is applied. Each individual is an ensemble of trees and is generated with the ramped half-and-half method as in [18]. With this method, an equal number of trees are initialized for each depth between 2 and the initial maximum tree depth value. For each depth level considered, half of the trees receive nonterminals from the function set until trees are fully grown; the other half is allowed to receive nodes from both the nonterminal and terminal sets randomly except for the root node, producing a group of heavily unbalanced trees. Then this method results in balanced and unbalanced trees with several different depths. The max depth of each individual is restricted to 3, and each nonterminal is forced to have exactly three children, which can be terminals or nonterminals. The crossover rate is 0.8, and the mutation rate is 0.4. The population size is 80, and the maximum number of generations is 200. The fitness function for each individual is its accuracy on the validation set (see Section 2.4). The implementation of GP is based on the Pyevolve library (0.6rc1) [28].

The population in the last generation is reserved for ensemble selection. Since each individual is already an ensemble classifier, the step of further fusing individuals of the final generation is a kind of metalearning. First, like the decision tree building phase (Section 2.2), we select individuals above the average accuracy of all the individuals as the available individuals. Second, a forward search algorithm [14] is applied: initially, the best individual is selected in the final committee. And then at each step, we iterate all pairs of the available individuals (not yet in the final committee) to find out the best pair that reduces the majority voting error (MVE) the most. Usually this step stops when all the individuals are exhausted or no improvement could be found. However, because there are only a small number of samples and each individual is usually a competent learner, it is found that this forward search process ceases after adding one or two pairs. As a result, we still take the risk of getting a low-bias and high-variance model. Based on this observation, it is necessary to include more individuals in the final ensemble to reduce the variance. So this algorithm is adapted to stop the forward search process when one of these conditions is satisfied: (1) a worse value of MVE is observed instead of no improvement; (2) all individuals are exhausted.

### 2.5. Cross Validation to Avoid Overfitting

For the aforementioned steps, we need to evaluate base classifiers' performances in various phases:

- selecting decision trees with above average accuracy of the 300 candidates,
- evaluating fitness values for individuals in each generation in the evolutionary process,
- selecting accurate individuals in the final generation and using the forward search algorithm to select proper members for the final committee.

One common approach to avoid overfitting in the training set is to split off part of it as an independent validation set. However, an algorithm can also overfit the validation set because of small sample size. To overcome this, in our method, we split the training set with 3-fold stratified cross validation and feed different folds for these three phases, respectively. For 3-fold cross validation, the data is randomly and evenly split into 3 groups. The evaluation takes 3 iterations. In each iteration, one of the 3 groups is selected in turn as a validation set, and the remaining 2 groups are selected as the training set. And in each group, the ratio of samples in the two classes is kept the same in both training and validation sets.

**Figure 3:**
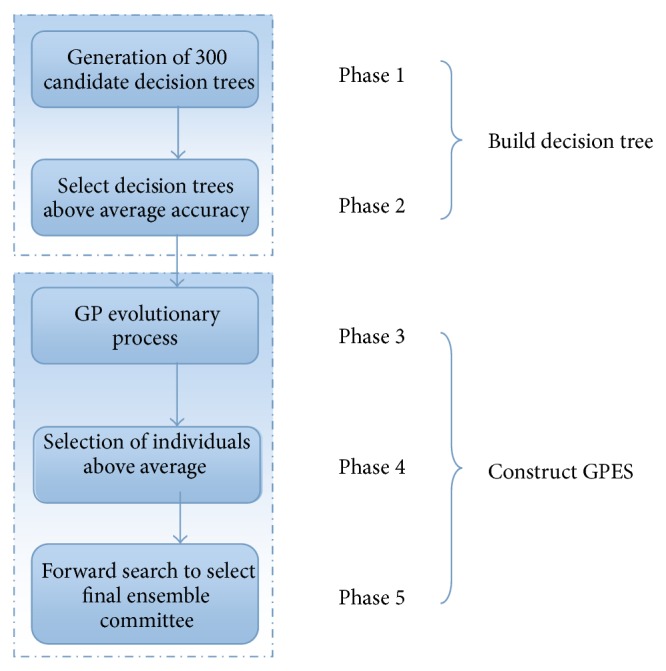
Decompose GPES into different phases.

We decompose the training process of GPES into five different phases, as shown in Figure 3. Phase 1 generates 300 candidate trees with random feature and sample subsets; Phase 2 selects accurate trees from the candidates. Phase 1 and Phase 2 consist of the decision tree building process, and they use the first training (Phase 1) set and the validation (Phase 2) set. Phase 3 deploys GP to evolve a group of candidates; Phase 4 selects accurate individuals from the population in the last generation; Phase 5 uses a forward search algorithm to select the final ensemble committee. In general, Phases 3–5 consist of the GP evolutionary process. Phase 3 uses the first training set for training base classifiers and the validation sets for calculating fitness value, while Phases 4 and 5 share the second training set and the validation set for retraining individuals in last generation, so as to realize the selection of above-average individuals and the forward search step efficiently.

## 3. Experimental Results and Analysis

To evaluate the effectiveness of GPES, we compare it with some tree-based learners, including decision tree, Random Forest, and Rotation Forest. They are evaluated on several binary and multiclass microarray datasets, as shown in Tables 1 and 2. For decision tree (both in standalone and GP) and Random Forest, the implementations in scikit-learn library (0.12) are used [24] with the default setting. For Rotation Forest, the implementation in Weka with the default setting is used. The standardization (Section 2.2.1) and the feature selection (Section 2.2.2) steps are applied for all the classifiers. In other words, all the classifiers receive the same reduced subset of features for a given training and test dataset. Besides them, a genetic algorithm (GA) based ensemble SVM learner based on gene pairs (GA-ESP) is also applied for comparison, with the same parameters in [29].

### 3.1. Results and Analysis on Binary Class Datasets

In this section, we present and analyze the results of GPES in five binary class microarray datasets. The detailed information about these datasets is listed in Table 1. Most of the original datasets are unbalanced. For example, for the Lung dataset, numbers of samples in the two classes of the training dataset are 134/15. So the ratio of two classes is close to 9/1, which makes the classification problem hard to deal with. As we use the subsample technique to obtain a balanced training set, the balanced training set consists of 15 samples for each class. As a result, the base classifiers of different ensemble systems tend to be of high diversity.

To evaluate the fitness function in the GP evolutionary process, 10-fold stratified cross validation is applied, based on the corresponding selected training set and validation set with the original division, as introduced in Section 2.4.

For unbalanced binary class problems, AUC is an effective measure besides accuracy, as discussed in [30]. After obtaining the values of true positive (TP), true negative (TN), false positive (FP), and false negative (FN), AUC is calculated by

(1) $$\begin{array}{c} \text{AUC}=\frac{1}{2}\left(\frac{\text{TP}}{\text{TP}+\text{FN}}+\frac{\text{TN}}{\text{TN}+\text{FP}}\right). \end{array}$$

Each algorithm runs 20 times with the same 10-fold split. For both accuracy and AUC, the corresponding average value and standard deviation on each test set are listed in Table 3. From the results shown in Table 3, we can see that GPES wins three cases, and both Rotation Forest and GA-ESP win two cases in all datasets. Random Forest and the single decision tree never achieve the best performance in all experiments. It can be found that when the base classifier is accurate enough, for example, in the case of Leukemia data, GPES can obtain 96.1% average accuracy, which is about 10% improvement compared with the accuracy of a single tree. In comparison of GPES, GA-ESP, and Rotation Forest, it should be noted that Rotation Forest uses PCA to transform the original features, so the base classifiers receive different inputs from the original feature subsets. On the contrary, GPES and GA-ESP can carry out the classification task in the original feature subspaces, which allows researchers to further investigate frequently selected genes. And the highest average accuracy is still achieved by GPES with the lowest deviation. It is obvious that GPES is able to improve the accuracy of decision tree and outperforms Random Forest in most cases.

**Figure 4:**
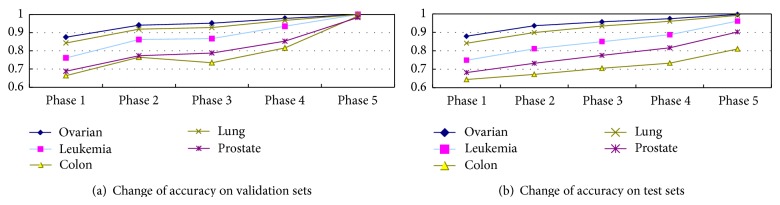
Change of accuracy in different phases.

Figure 4 shows the average accuracy of GPES in different phases (see Section 3.2) in a run, on both validation sets and test sets. Note that the samples of test sets are never mixed with the training set, so the results are reliable. The changes of the accuracy in different phases on validation sets are plotted in Figure 4(a). It can be found that Phase 2 boosts the performances compared with those in Phase 1 significantly, because its functionality is to select above-average trees in the validation set. The transition from Phase 2 to Phase 3 is the GP evolutionary process. As Phase 3 represents the results of the last generation of GP, the average performances of individuals become better and better. So it can be observed that the curves ascend stably in most cases. Colon dataset is an exception. As can be found in Figure 4(a), the curve drops in Phase 3. The reason may lie in that, in Phase 3, a new training/validation set is used. The sample size of original dataset is too small, and the training set and validation set are not in the same distribution. As a result, the trained classifiers cannot fit the validation set well. Phase 4 effectively raises the curve again because only the individuals above the average performance are kept. Then, in the final forward search step (Phase 5), the accuracy curves raise a lot again for all datasets.

The changes of average accuracy in test sets are plotted in Figure 4(b). Independent test sets are also be inputted in different phases, so that variation of the accuracy on test sets in these different phases can also be evaluated. Overall, the performance on the test sets keeps increasing in each phase for all different datasets. And it is observed that even for the Colon dataset, whose average curve drops in Phase 3 in validation set, the curve keeps going up in the test set. This is an evidence that GPES does not overfit in these datasets.

**Figure 5:**
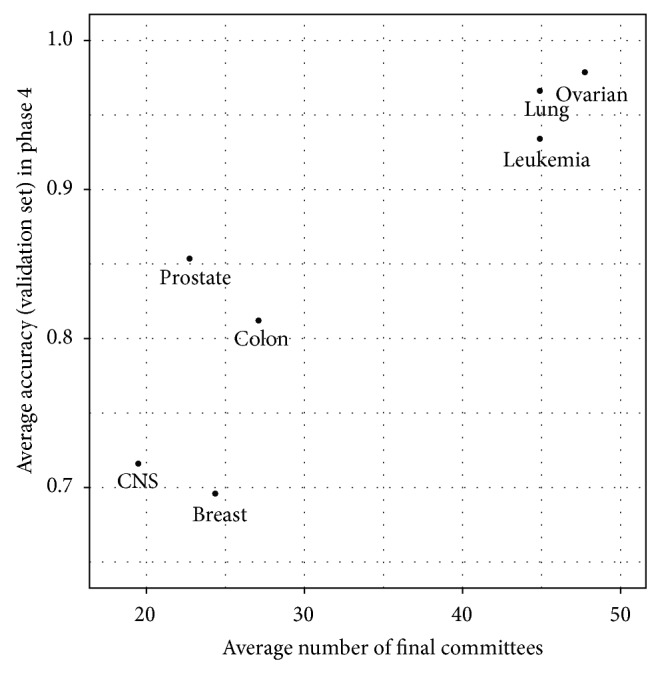
Average accuracy (validation set) in Phase 4 versus average number of final ensemble systems.

Figure 5 shows the plot of average accuracy (on validation set) in Phase 4 versus average number of individuals in the final ensemble committee. It is clear that the number of individuals in the final ensemble committee is roughly correlated with the average accuracy in Phase 4. That is, when the average accuracy is high, the number of individuals included in the final ensemble committee is large. It is desirable in the task of microarray classification. Because when the accuracy of base classifiers is high, incorporating more classifiers can help to reduce variance. On the contrary, when the average accuracy is low, it is usually hard to find a lot of reliable (accurate) candidates, and the combination of more classifiers will not benefit the final results. Thus, the algorithm is able to adaptively adjust the size of the final ensemble committee according to the characteristics of the dataset.

Table 4 lists the average percentages of different operators appearing in the final ensemble committee for different datasets. In all the cases, the Average operator appears with the highest frequency, and the distributions of different operators are different among different datasets. For example, the percentages of Min operators are relatively high for the Colon and Lung datasets, while low for the Prostate dataset. It is obvious that the application of diverse operators contributes to the effectiveness of the final ensemble committee.

### 3.2. Results and Analysis on Multiclass Datasets

In the following experiments, six multiclass microarray datasets are deployed to evaluate the performance of GPES in multiclass problems, and their detailed information is listed in Table 2.

For a *m*-class problem, different class labels are represented as 1,2,…, *m*. The base classifiers used in our experiments are decision trees, and they can only output hard class labels, which are used to indicate corresponding different classes. So it is meaningless to apply the Average, Min, and Max operators to different class labels directly. In order to make all operators work effectively, a multiclass problem is decomposed to a set of binary class problems in the following experiments. Two commonly used decomposition methods are employed: one versus one (OVO) and one versus rest (OVR). By this mean, despite the fact that the decision tree can deal with multiclass problem directly, it is only used as a binary classifier in the following experiments. For fair comparisons, decision tree, Random Forest, and Rotation Forest methods are also used as binary classifiers, fused with OVO and OVR methods. And it should be noted that decision trees are also ensemble systems after they are fused with OVO and OVR methods in experiments. The Rotation Forest algorithm used in binary class problems is based on Weka software, and it is not easy for us to feed the decomposed data to the Rotation Forest function in the software. So we use an improved Rotation Forest algorithm and hybrid extreme rotation forest (HERF) [31] in the experiments for multiclass problems instead. The python implementation available at [32] is used with the default setting.

The framework of GPES is the same as that for the binary class problem, and it is still set to contain three children for each operator so as to control the size of ensemble system. And we use two different measures, *F*score_*μ*_ and average accuracy (AA*c* for short) [30], for results comparisons. Assume that there are *c* classes in a dataset, and then these two measures can be calculated by formulas (2)–(5):

(2) Precisionμ=∑i=1ctpi∑i=1ctpi+fpi,

(3) Recallμ=∑i=1ctpi∑i=1ctpi+fni,

(4) Fscoreμ=β2+1PrecisionμRecallμβ2Precisionμ+Recallμ,

(5) AAc=∑i=1ctpi+tni/tpi+tni+fpi+fnic.

Different from accuracy, AA*c* indicates the average per-class performance of a classifier. If a classifier fails to recognize samples in a “hard” class, it cannot achieve high scores in AA*c*. *F*score_*μ*_ is a measure combining the scores of both precision and recall. To get a balance between precision and recall, *β* is set to 1 for formula (4).

Results obtained by different methods are listed in Table 5. Among the twelve groups of results, it is obvious that GPES (OVO) wins four cases; GA-ESP (OVR) wins three cases; GPES (OVR), Random Forest (OVO), and HARF (OVO) win two cases. In addition, GPES (OVO) achieves the highest average accuracy with low variance for both measures.

The performance of GPES (OVR) is worse than GPES (OVO) in most cases, especially on Breast and DLBCL datasets. The failure of the OVR scheme is mainly caused by the undersampling technique. When the number of classes is large enough, for example, 6 classes in DLBCL dataset, this undersampling technique receives two different parts of samples to construct the training dataset in OVR scheme: the first part from a class and the second part from the remaining 5 classes. To get balanced datasets, the size of the second part is the same as that of the first part with undersampling technique. Since for the fourth binary problem (to distinguish the fourth class from other classes), the number of the fourth class in the training dataset is 4, the number of the second part for the training dataset must be set to 4. It is a quite small value, which does not allow the training set to obtain even a sample from each of the remaining five classes. As a result, this scheme faces the problem of a severely insufficient training sample in this case, and the base classifier cannot fully learn the distribution of the two parts. So, it can be expected that when applying other sampling techniques, the performance of GPES with OVR would be boosted. However, since its score is only 1% lower than that of Random Forest with OVR in both measures, and its average performances are still ranked as the fourth best method in the experiments, we do not further explore the application of different subsample techniques.

It should be noted that the average variances of the GPES and GA-ESP methods are lower than those of Random Forest and HARF methods, which indicates the relatively stable performances of evolutionarily based ensemble methods in multiclass problems. As the multiclass problem is the combination of a set of binary problems, the observations in these experiments are similar with those of binary class problems.

## 4. Conclusion

In this paper, we propose a new GP based ensemble system, named as GPES. Decision trees are deployed as base classifiers in this ensemble framework with three operators: Min, Max, and Average. The evolutionary process makes the ensemble system be adapted to better solve the classification problem for both binary class and multiclass microarray datasets.

The training process is carefully designed to inject diversity at feature, sample, and base classifier levels. In this way, the final ensemble committee is composed of accurate and diverse base classifiers, so it can effectively avoid overfitting. The effectiveness of GPES is evaluated in five binary class and six multiclass microarray datasets. It is found that the algorithm is proved to be adaptive to the characteristics of datasets. In addition, although the base classifier of this algorithm is decision tree, other classification models can also be used as base classifiers, such as neural networks and *k*-nearest neighbor. By using elaborate base classifiers, or applying other sampling techniques, the performance of GPES may be further improved.

## Figures and Tables

**Table 1:** Binary class datasets used in experiments.

Datasets	Number of genes	Number of samples of two classes	Reference
Ovarian	15154	162/91	[33]
Leukemia	7129	47/25	[34]
Colon	20000	40/22	[35]
Lung	12533	150/31	[36]
Prostate	12600	77/59	[37]

**Table 2:** Multiclass datasets used in experiments.

Dataset	Number of classes	Number of genes	Number of training samples	Number of test samples	Reference
Leukemia 1	3	7129	38	34	[34]
Leukemia 2	3	12,582	57	15	[22]
Lung 1	3	7129	64	32	[38]
Lung 2	5	12,600	136	67	[39]
Breast	5	9216	54	30	[40]
DLBCL	6	4026	58	30	[41]

**Table 3:** Experimental results for binary datasets.

Datasets	GPES	DT	Random Forest	Rotation Forest	GA-ESP
Ovarian					
Accuracy	**0.997 ± 0.003**	0.984 ± 0.000	0.988 ± 0.006	**0.997 ± 0.003**	**0.997 ± 0.003**
AUC	**0.992 ± 0.004**	0.987 ± 0.000	0.990 ± 0.000	**0.992 ± 0.004**	**0.992 ± 0.004**
Leukemia					
Accuracy	**0.961 ± 0.013**	0.863 ± 0.000	0.944 ± 0.016	0.934 ± 0.020	0.944 ± 0.012
AUC	**0.972 ± 0.012**	0.829 ± 0.000	0.942 ± 0.006	0.945 ± 0.017	0.935 ± 0.010
Colon					
Accuracy	0.810 ± 0.024	0.743 ± 0.000	0.804 ± 0.030	0.820 ± 0.025	**0.830 ± 0.026**
AUC	**0.805 ± 0.017**	0.756 ± 0.000	0.795 ± 0.024	0.796 ± 0.022	**0.805 ± 0.013**
Lung					
Accuracy	**0.992 ± 0.004**	0.967 ± 0.000	0.985 ± 0.005	0.990 ± 0.005	0.980 ± 0.009
AUC	**0.988 ± 0.003**	0.927 ± 0.000	0.980 ± 0.003	0.986 ± 0.004	0.963 ± 0.007
Prostate					
Accuracy	0.902 ± 0.014	0.889 ± 0.000	0.889 ± 0.017	**0.912 ± 0.020**	0.890 ± 0.018
AUC	0.889 ± 0.010	0.831 ± 0.000	0.862 ± 0.011	**0.891 ± 0.013**	0.885 ± 0.015

Average					
Accuracy	**0.932 ± 0.011**	0.889 ± 0.000	0.837 ± 0.020	0.930 ± 0.015	0.928 ± 0.014
AUC	**0.929 ± 0.009**	0.866 ± 0.000	0.914 ± 0.009	0.922 ± 0.012	0.905 ± 0.010

**Table 4:** Percentage of different operators in the final committee.

Datasets	% of Min	% of Average	% of Max
Ovarian	0.257	0.567	0.177
Leukemia	0.240	0.567	0.194
Colon	0.374	0.449	0.177
Lung	0.333	0.487	0.180
Prostate	0.178	0.582	0.240

**Table 5:** Experimental results for multiclass datasets.

Datasets	OVO					OVR				
	GPES	DT	Random Forest	HARF	GA-ESP	GPES	DT	Random Forest	HARF	GA-ESP
Leukemia 1										
*F*score_*μ*_	0.897 ± 0.006	0.941 ± 0.000	0.881 ± 0.061	0.923 ± 0.074	0.932 ± 0.010	**0.965 ± 0.017**	0.882 ± 0.000	0.917 ± 0.012	0.941 ± 0.019	0.903 ± 0.012
AAc	0.905 ± 0.004	0.960 ± 0.000	0.918 ± 0.040	0.949 ± 0.049	0.961 ± 0.007	**0.976 ± 0.001**	0.922 ± 0.000	0.944 ± 0.008	0.951 ± 0.012	0.938 ± 0.019
Leukemia 2										
*F*score_*μ*_	0.933 ± 0.042	0.733 ± 0.000	0.934 ± 0.066	**0.956 ± 0.011**	0.935 ± 0.018	0.891 ± 0.046	0.773 ± 0.000	0.921 ± 0.050	0.925 ± 0.026	0.902 ± 0.014
AAc	0.956 ± 0.025	0.778 ± 0.000	0.953 ± 0.043	**0.997 ± 0.008**	0.978 ± 0.016	0.925 ± 0.028	0.882 ± 0.000	0.947 ± 0.033	0.940 ± 0.017	0.922 ± 0.008
Lung 1										
*F*score_*μ*_	**0.829 ± 0.029**	0.668 ± 0.000	0.791 ± 0.017	0.817 ± 0.013	0.812 ± 0.025	0.822 ± 0.033	0.781 ± 0.000	0.771 ± 0.025	0.818 ± 0.036	0.802 ± 0.013
AAc	**0.892 ± 0.024**	0.792 ± 0.000	0.861 ± 0.011	0.869 ± 0.009	0.853 ± 0.013	0.881 ± 0.023	0.854 ± 0.000	0.847 ± 0.017	0.868 ± 0.026	0.865 ± 0.012
Lung 2										
*F*score_*μ*_	0.948 ± 0.030	0.955 ± 0.000	0.950 ± 0.008	0.933 ± 0.013	0.942 ± 0.021	0.913 ± 0.013	0.851 ± 0.000	0.908 ± 0.014	0.946 ± 0.017	**0.957 ± 0.013**
AAc	0.979 ± 0.012	0.982 ± 0.000	0.980 ± 0.031	0.965 ± 0.009	0.953 ± 0.016	0.960 ± 0.007	0.940 ± 0.000	0.963 ± 0.006	0.964 ± 0.013	**0.988 ± 0.014**
Breast										
*F*score_*μ*_	0.875 ± 0.026	0.700 ± 0.000	**0.888 ± 0.027**	0.860 ± 0.037	0.853 ± 0.020	0.821 ± 0.004	0.733 ± 0.000	0.844 ± 0.027	0.873 ± 0.037	**0.888 ± 0.020**
AAc	0.947 ± 0.014	0.880 ± 0.000	**0.961 ± 0.002**	0.946 ± 0.012	0.916 ± 0.009	0.915 ± 0.003	0.893 ± 0.000	0.949 ± 0.017	0.942 ± 0.015	0.912 ± 0.010
DLBCL										
*F*score_*μ*_	**0.967 ± 0.019**	0.833 ± 0.000	0.935 ± 0.055	0.837 ± 0.007	0.932 ± 0.010	0.883 ± 0.032	0.833 ± 0.000	0.815 ± 0.071	0.803 ± 0.071	0.922 ± 0.013
AAc	**0.989 ± 0.006**	0.944 ± 0.000	0.972 ± 0.018	0.925 ± 0.024	0.958 ± 0.007	0.947 ± 0.011	0.944 ± 0.000	0.937 ± 0.023	0.923 ± 0.023	0.965 ± 0.009

Average										
*F*score_*μ*_	**0.908 ± 0.025**	0.805 ± 0.000	0.897 ± 0.039	0.888 ± 0.026	0.901 ± 0.017	0.882 ± 0.024	0.809 ± 0.000	0.863 ± 0.033	0.884 ± 0.034	0.896 ± 0.014
AAc	**0.945 ± 0.014**	0.889 ± 0.000	0.941 ± 0.024	0.942 ± 0.018	0.937 ± 0.013	0.934 ± 0.012	0.906 ± 0.000	0.878 ± 0.055	0.931 ± 0.017	0.932 ± 0.012
